# Synchronization and vibratory synchronization transmission of a weakly damped far-resonance vibrating system

**DOI:** 10.1371/journal.pone.0209703

**Published:** 2019-03-25

**Authors:** Bang Chen, Xiao’ou Xia, Xiaobo Wang

**Affiliations:** 1 School of Mechanical Engineering, University of Science and Technology Beijing, Beijing, China; 2 BGRIMM Technology Group, Beijing, China; Lanzhou University of Technology, CHINA

## Abstract

The self-synchronization of rotors mounted on different vibrating bodies can be easily controlled by adjusting the coupling parameters. To reveal the synchronization characteristics of a weakly damped system with two rotors mounted on different vibrating bodies, we propose a simplified physical model. The topics described in this paper are related to coupling dynamic problems between two vibrating systems. Both synchronization and vibratory synchronization transmission of the system are studied. The coupling mechanism between the two rotors is analyzed to derive the synchronization condition and the stability criterion of the system. The vibration of the system is described by an averaging method that can separate fast motion (high frequency) from slow motion (low frequency). Theoretical research shows that vibration torque is the key factor in balancing the energy distribution between the rotors. Taking the maximum vibration torque (MVT) as a critical parameter, we investigate the synchronization characteristics of the vibrating system in different cases. The curve of the maximum vibration torque (MVT) versus coupling frequency is divided into several parts by the coupling characteristic frequency and the input torque difference between the rotors. Simulations of the system with coupling frequencies from different parts are carried out. For the system with rotational frequencies larger than the natural frequencies, the coupling characteristic frequency or characteristic frequency curve should be considered. When the coupling frequency is close to the characteristic frequency or the vibration state is close to the characteristic frequency curve, self-synchronization of the two rotors can be obtained easily. Under certain conditions when the coupling effect between the rotors is strong enough, the rotors can maintain synchronous rotation even when one of the two motors is shut off after synchronization is achieved, which is called vibratory synchronization transmission. Vibratory synchronization transmission of the system occurs in a new synchronous condition, and the phase difference between the rotors takes on a new value, that is, the system approaches a new synchronization state.

## Introduction

In addition to chaos, synchronization is an important concept in the research of nonlinear vibration. In recent years, many researchers and engineers have devoted their efforts to studying synchronization phenomena in different fields.

The so-called self-synchronization phenomenon corresponds to the consistency or a particular relationship between systems’ parameters resulting from internal couplings. Sometimes, synchronization is called frequency capture, which indicates that the frequencies of different vibrations synchronize. Vibration self-synchronization has been widely relevant in nonlinear vibration, hydraulic [1–3], electromechanical coupling, automatic control theory and other fields [4–8].

Huygens was the first person to observe and study the synchronization of pendulum clocks hanging from a common moving frame in the 17th century. The works of PEÑA, Jovanovic and Dilao indicated [5–7] the effects of parameters on synchronization of pendula both in phase and out of phase. In [8–10], Czolczynski et al. presented different synchronous behavior of two or *n* pendula installed on a frame. Zhang et al. [11] concentrated on metronome synchronization on two layers in an asymmetric coupling scheme. Koluda [12–13] suggested that four synchronous configurations could be obtained by two self-excited double pendula mounted on a moving beam. The achievements of scholars constantly enrich the existing research on the synchronization of pendula [7, 14–16]. The above studies are mainly about oscillations of pendula. The rotational motions of the pendula have also attracted much attention recently due to the concept of harnessing energy from sea waves [17]. Czolczynski and Strzalko [18–19] described the synchronous rotation of pendula installed on a vibrating frame. Experiments with four double-coupled pendula were carried out by Dawid [20], which suggested that multistable states could be observed in small networks of coupled pendula. The self-synchronization theory of rotors was developed by Blekhman [1–2, 21] with the averaging method, and the synchronization conditions and stability of the vibrating system were summarized in the middle of the 20th century. The averaging method is commonly used in solving nonlinear vibration problems. It can directly separate the high-frequency motion of the system from the low-frequency motion and simplify the analysis. Wen and Zhao et al. [22–24] modified the averaging method by introducing two variable perturbation parameters and proposed two small parameters to average the angular velocity of the two exciters and their phase difference. This method is useful for investigating the synchronization of a vibrating system with two nonidentical coupled exciters, and the dynamic characteristics of the induction motor are more involved. Zhang [25, 26] deduced the synchronization condition and the synchronization stability for the vibrating system with three rotors. Hou and Fang [27–29] investigated a vibrating screen based on the model of a rotor-pendulum system and clarified the synchronization condition and stability of the system by the Poincare method.

The above studies were mostly focused on the synchronization of pendula or rotors installed on the same vibrating frame. It was found that the coupling performance of two rotors mounted on the same vibrating body was not the strongest and that the setup is not suitable for some heavy-load and high-impact conditions. Aiming at this problem, we propose a vibrating system with two rotors mounted on two coupling bodies. In the system, two vibrating bodies are connected by a coupling spring. The stability of the system can be enhanced by controlling the coupling spring. In addition, it is convenient to control the synchronous performance of the system. In this paper, we explore the influence of the coupling spring stiffness on the dynamics of the system and establish a relationship between synchronous performance and spring stiffness, which can provide a theoretical basis for the design of high-stability and high-tolerance systems.

The paper is organized as follows. The strategy used in this paper is introduced in section 2. In section 3, the considered model is described, and the differential equations of the vibrating system are given. Section 4 presents the analytical studies on the self-synchronization equation and the stability criterion of the system. The influence of the coupling spring stiffness on synchronization is explored in section 5. Numerical simulations of the system with different parameters are carried out to verify the theoretical analysis in Sections 6 and 7, and section 8 shows our conclusions.

## Strategy

The dynamics of the considered rotational system can be expressed as follows:

$$\begin{array}{l} J_{s}{\ddot{\varphi }}_{s}=\mu \Phi_{s}\left(\varphi_{s},{\dot{\varphi }}_{s},{\ddot{\varphi }}_{s},x,\dot{x},\ddot{x}\right)\\ \ddot{x}+c_{x}\dot{x}+k_{x}x=\sum_{i=1}^{k}T_{sx} \end{array}$$

where *φ*_*s*_ is the rotation angle of the s-th rotor, and *x* is the displacement of the moving body. In the expression *μΦ*_*s*_ *= T*_*es–*_*T*_*fs*_*+T*_*xs*_, *μ* is a small parameter, *J*_*s*_ is the rotational inertia of the *s*-th rotor, and *T*_*es*_, *T*_*fs*_ are the driving torque and damping torque of the s-th motor, respectively. *T*_*xs*_, *T*_*sx*_ correspond to the coupling effects between the s-th rotors and the moving body.

In the synchronous state, the synchronous velocity of the system is *ω*_*n*_. Thus, *φ*_*s*_ can be expressed by *φ*_*s*_ = *ω*_*n*_*t+α*_*s*_, where *α*_*s*_ is the phase of the s-th rotor. Self-synchronization of the rotors can only be obtained when the resultant torque acting on the s-th rotor is equal to 0 in one vibration period of the system, and we have

$$P_{s}\left(\alpha_{1},\cdots ,\alpha_{k}\right)=\frac{1}{T}\int_{0}^{T}\mu \Phi_{s}dt=0$$ (1)

*α*_*s*_ can be deduced by Eq (1). The stability of the synchronization system can be described by the following equation:

$$\left|\begin{array}{cccc} \frac{\partial P_{1}}{\partial \alpha_{1}}-\lambda & \frac{\partial P_{1}}{\partial \alpha_{2}} & \cdots & \frac{\partial P_{1}}{\partial \alpha_{k}}\\ \frac{\partial P_{2}}{\partial \alpha_{1}} & \frac{\partial P_{2}}{\partial \alpha_{2}}-\lambda & \cdots & \frac{\partial P_{2}}{\partial \alpha_{k}}\\ \vdots & \vdots & \ddots & \vdots \\ \frac{\partial P_{k}}{\partial \alpha_{1}} & \frac{\partial P_{k}}{\partial \alpha_{2}} & \cdots & \frac{\partial P_{k}}{\partial \alpha_{k}}-\lambda \end{array}\right|=0$$ (2)

Only if all the solutions of Eq (2) have a negative component does the synchronization of rotors become stable. However, if one of the solutions of Eq (2) has a positive component, the corresponding synchronous rotation of the rotors is unstable. For zero or imaginary solutions, further analysis should be carried out.

## Dynamical equations of the vibrating system

**Fig 1:**
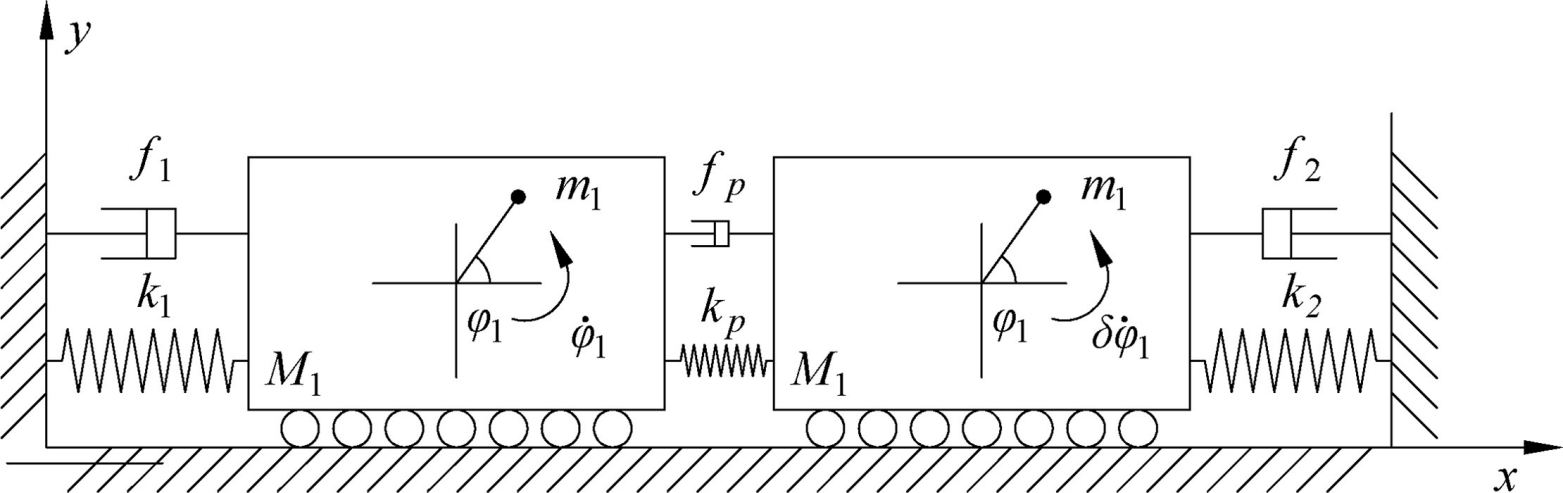
Simplified model of the system.

As shown in Fig 1, two rotors are mounted on two different bodies. The two bodies are connected by a coupling spring, and the stiffness and damping coefficient are denoted by *k*_*p*_, *f*_*p*_, respectively. The masses of the rotors are *m*_*1*_ and *m*_*2*_. The phase of each rotor rotating about its spin axis is given by *φ*_*i*_ (*i* = 1). The electromagnetic torque and resistance moment of the driving motor are *T*_*ei*_ and *T*_*fi*_, respectively, and the resistance coefficient of the motor is described by *f*_*ri*_. The counterclockwise direction is taken to be positive. The vibrating body (*M*_*i*_) can move in the horizontal direction (*x*_*i*_) and is installed on the foundation by the spring characterized by stiffness coefficient *k*_*i*_ and damping coefficient *f*_*i*_. The inertia moment and eccentricity of the rotor on its mass center are given by *j*_*i*_ and *r*_*i*_, respectively. In this paper, synchronization of the rotors is analyzed in a nonresonant vibrating system, in which the rotation frequencies of the rotors are larger than the natural frequencies of the vibrating bodies, and the system is referred to as an after-resonance system. We assume that

$${\dot{\varphi }}_{1},{\dot{\varphi }}_{2}>2\omega_{1},2\omega_{2}$$

where $\omega_{1}^{2}$ = *k*_*1*_*/M*_*1*_, and $\omega_{2}^{2}$ = *k*_*2*_*/M*_*2*_.

Differential equations can be derived by Lagrange’s equations. The kinetic energy of the system is expressed as

$$\begin{array}{l} T=\frac{1}{2}M_{1}{\dot{x}}_{1}^{2}+\frac{1}{2}M_{2}{\dot{x}}_{2}^{2}\\ +\frac{1}{2}m_{1}\left[{\left({\dot{x}}_{1}-{\dot{\varphi }}_{1}r_{1}\sin\varphi_{1}\right)}^{2}+{\left({\dot{\varphi }}_{1}r_{1}\cos\varphi_{1}\right)}^{2}\right]\\ +\frac{1}{2}m_{2}\left[{\left({\dot{x}}_{2}-\delta {\dot{\varphi }}_{2}r_{2}\sin\varphi_{2}\right)}^{2}+{\left(\delta {\dot{\varphi }}_{2}r_{2}\cos\varphi_{2}\right)}^{2}\right]\\ +\frac{1}{2}j_{1}{\dot{\varphi }}_{1}^{2}+\frac{1}{2}j_{2}{\dot{\varphi }}_{2}^{2}. \end{array}$$

where *δ* = 1 (in the anticlockwise direction) or -1 (in the clockwise direction). The potential energy and the dissipative function can be described as follows:

$$V=\frac{1}{2}k_{1}x_{1}^{2}+\frac{1}{2}k_{2}x_{2}^{2}+\frac{1}{2}k_{p}{\left(x_{1}-x_{2}\right)}^{2}.$$

$$D=\frac{1}{2}f_{1}{\dot{x}}_{1}^{2}+\frac{1}{2}f_{2}{\dot{x}}_{2}^{2}+\frac{1}{2}f_{p}{\left({\dot{x}}_{1}-{\dot{x}}_{2}\right)}^{2}.$$

The generalized forces acting on the vibrating bodies are zero, and the generalized forces acting on rotors 1 and 2 are *T*_*e1–*_*T*_*f1*_ and *T*_*e2–*_*T*_*f2*_, respectively. When the system operates stably and the speed of the rotor d*φ*_*i*_/d*t* (*i* = 1,2) fluctuates near the frequency *ω*_*n*_, the influence of electromagnetic leakage is neglected, and the driving force of the induction motor can be linearized as [24, 25, 29]

$$T_{ei}=n\frac{L_{mi}^{2}U_{0}^{2}}{L_{si}^{2}\omega_{si}R_{ri}}\cdot \frac{\omega_{si}-n\omega_{n}}{\omega_{si}}.$$

where *n*_*i*_, *L*_*mi*_, *ω*_*si*_, *L*_*si*_ and *R*_*ri*_ (*i* = 1,2) correspond to the pole number, mutual inductance, synchronous speed, stator inductance and rotor resistance of the motor, respectively; *U*_*0*_ is the voltage amplitude. The vibration differential equations of the system are described as follows:

$$\begin{array}{l} M_{1}{\ddot{x}}_{1}+f_{1}{\dot{x}}_{1}+k_{1}x_{1}+k_{p}\left(x_{1}-x_{2}\right)+f_{p}\left({\dot{x}}_{1}-{\dot{x}}_{2}\right)=m_{1}r_{1}\left({\ddot{\varphi }}_{1}\sin\varphi_{1}+{\dot{\varphi }}_{1}^{2}\cos\varphi_{1}\right)\\ M_{2}{\ddot{x}}_{2}+f_{2}{\dot{x}}_{2}+k_{2}x_{2}+k_{p}\left(x_{2}-x_{1}\right)+f_{p}\left({\dot{x}}_{2}-{\dot{x}}_{1}\right)=\delta m_{2}r_{2}\left({\ddot{\varphi }}_{2}\sin\varphi_{2}+{\dot{\varphi }}_{2}^{2}\cos\varphi_{2}\right)\\ \left(j_{1}+m_{1}r_{1}^{2}\right){\ddot{\varphi }}_{1}=T_{e1}-T_{f1}+m_{1}r_{1}{\ddot{x}}_{1}\sin\varphi_{1}\\ \left(j_{2}+m_{2}r_{2}^{2}\right){\ddot{\varphi }}_{2}=T_{e2}-T_{f2}+\delta m_{2}r_{2}{\ddot{x}}_{2}\sin\varphi_{2} \end{array}$$

## Synchronization and stability of the system

In this section, the approximate analytical self-synchronization equation and the stability criterion of the system are derived. The model in this paper is a vibration system with stiffness coupling. As self-synchronization of the rotors is achieved in a nonresonant vibrating system, the system can run stably, and the speed fluctuations of the rotors are small [1, 2]. The damping coefficients of the springs in the system are considered to be very small in the actual industrial application of interest, that is, a weakly damped system with two rotors [1]. Neglecting small variables, we have

$$\begin{array}{l} {\ddot{x}}_{1}+\omega_{1}^{2}x_{1}+\eta \omega_{p}^{2}\left(x_{1}-x_{2}\right)=\frac{m_{1}}{M_{1}}r_{1}{\dot{\varphi }}_{1}^{2}\cos\varphi_{1}\\ {\ddot{x}}_{2}+\omega_{2}^{2}x_{2}+\omega_{p}^{2}\left(x_{2}-x_{1}\right)=\frac{m_{2}}{M_{2}}\delta r_{2}{\dot{\varphi }}_{2}^{2}\cos\varphi_{2}\\ J_{1}{\ddot{\varphi }}_{1}=T_{e1}-T_{f1}+m_{1}r_{1}{\ddot{x}}_{1}\sin\varphi_{1}\\ J_{2}{\ddot{\varphi }}_{2}=T_{e2}-T_{f2}+\delta m_{2}r_{2}{\ddot{x}}_{2}\sin\varphi_{2} \end{array}$$ (3)

where

$$\begin{array}{l} \omega_{p}=\sqrt{k_{p}/M_{1}},\;\eta =M_{2}/M_{1},\\ J_{1}=j_{1}+m_{1}r_{1}^{2},\;J_{2}=j_{2}+m_{2}r_{2}^{2}. \end{array}$$

The synchronous speed of the two rotors is denoted by *ω*_*n*_. According to section 2, the synchronous speed is far from the natural frequency of the vibrating bodies. When self-synchronization of the rotors is achieved [1, 3], the phases of the two rotors can be written as follows:

$$\varphi_{1}=\omega_{n}t+\alpha_{1},\;\varphi_{2}=\omega_{n}t+\alpha_{2}.$$ (4)

where *α*_*1*_ and *α*_*2*_ are slowly varying parameters. From Eqs (3) and (4), we obtain

$$\begin{array}{l} x_{1}=\mu_{11}\cos\varphi_{1}+\mu_{12}\cos\varphi_{2},\\ x_{2}=\mu_{21}\cos\varphi_{1}+\mu_{22}\cos\varphi_{2}. \end{array}$$ (5)

where

$$\begin{array}{l} \mu_{11}=\frac{m_{1}r_{1}\omega_{n}^{2}\left(\omega_{2}^{2}+\omega_{p}^{2}-\omega_{n}^{2}\right)}{M_{1}\left[\left(\omega_{1}^{2}+\eta \omega_{p}^{2}-\omega_{n}^{2}\right)\left(\omega_{2}^{2}+\omega_{p}^{2}-\omega_{n}^{2}\right)-\eta \omega_{p}^{4}\right]},\mu_{12}=\frac{\delta m_{2}r_{2}\omega_{n}^{2}\omega_{p}^{2}}{M_{1}\left[\left(\omega_{1}^{2}+\eta \omega_{p}^{2}-\omega_{n}^{2}\right)\left(\omega_{2}^{2}+\omega_{p}^{2}-\omega_{n}^{2}\right)-\eta \omega_{p}^{4}\right]},\\ \mu_{21}=\frac{m_{1}r_{1}\omega_{n}^{2}\omega_{p}^{2}}{M_{1}\left[\left(\omega_{1}^{2}+\eta \omega_{p}^{2}-\omega_{n}^{2}\right)\left(\omega_{2}^{2}+\omega_{p}^{2}-\omega_{n}^{2}\right)-\eta \omega_{p}^{4}\right]},\mu_{22}=\frac{\delta m_{2}r_{2}\omega_{n}^{2}\left(\omega_{1}^{2}+\eta \omega_{p}^{2}-\omega_{n}^{2}\right)}{M_{2}\left[\left(\omega_{1}^{2}+\eta \omega_{p}^{2}-\omega_{n}^{2}\right)\left(\omega_{2}^{2}+\omega_{p}^{2}-\omega_{n}^{2}\right)-\eta \omega_{p}^{4}\right]}. \end{array}$$ (6)

*μ*_*11*_, *μ*_*12*_, *μ*_*21*_ and *μ*_*22*_ describe the coupling effects in the system. Obviously, the larger *μ*_*12*_ and *μ*_*21*_ are, the stronger the coupling between the rotors will be.

*μ*_*11*_ indicates the coupling coefficient between rotor 1 and vibrating body 1. *μ*_*12*_ indicates the coupling coefficient between rotor 2 and vibrating body 1. *μ*_*21*_ indicates the coupling coefficient between rotor 1 and vibrating body 2. *μ*_*22*_ indicates the coupling coefficient between rotor 2 and vibrating body 2.

It is a common approach to analyze nonlinear vibration problems by using the averaging method to separate fast motion (high frequency) from slow motion (low frequency). We define an operation such that if *h(t)* is a periodic function, its average value on a period of *T* is denoted as

$$\langle h\left(t\right)\rangle =\int_{t}^{t+T}h\left(t\right)\mathrm{dt}.$$ (7)

When the rotors rotate synchronously, the average acceleration of the rotor is 0, i.e.,

$$\begin{array}{l} \langle J_{1}{\ddot{\varphi }}_{1}\rangle =\langle T_{e1}-T_{f1}+m_{1}r_{1}{\ddot{x}}_{1}\sin\varphi_{1}\rangle =0,\\ \langle J_{2}{\ddot{\varphi }}_{2}\rangle =\langle T_{e2}-T_{f2}+\delta m_{2}r_{2}{\ddot{x}}_{2}\sin\varphi_{2}\rangle =0. \end{array}$$ (8)

The rotors are driven by motors, and the resistance of rotors is approximately proportional to their speed. When the rotor rotates steadily, the speed fluctuations are small. According to [16, 25], the output torque of the induction motor can be linearized. Therefore, <*T*_*ei*_(*ω*)>, <*T*_*fi*_(*ω*)> in Eq (8) can be replaced with *T*_*ei*_(*ω*_*n*_), *T*_*fi*_(*ω*_*n*_).

As the system runs stably, the average values of the resultant torques of rotors are denoted by *P*_*1*_ and *P*_*2*_. We have

$$\begin{array}{l} P_{1}=\langle J_{1}{\ddot{\varphi }}_{1}\rangle =\langle T_{e1}-T_{f1}+m_{1}r_{1}{\ddot{x}}_{1}\rangle =T_{e1}\left(\omega_{n}\right)-T_{f1}\left(\omega_{n}\right)+m_{1}r_{1}\langle {\ddot{x}}_{1}\sin\varphi_{1}\rangle =\\ T_{e1}\left(\omega_{n}\right)-T_{f1}\left(\omega_{n}\right)+m_{1}r_{1}\langle \left[-\mu_{11}\omega_{n}^{2}\cos\varphi_{1}-\mu_{12}\omega_{n}^{2}\cos\varphi_{2}\right]\sin\varphi_{1}\rangle \\ =T_{e1}\left(\omega_{n}\right)-T_{f1}\left(\omega_{n}\right)-\frac{1}{2}m_{1}r_{1}\mu_{12}\omega_{n}^{2}\sin\left(\alpha_{1}-\alpha_{2}\right), \end{array}$$ (9)

$$\begin{array}{l} P_{2}=\langle J_{2}{\ddot{\varphi }}_{2}\rangle =\langle T_{e2}-T_{f2}+\delta m_{2}r_{2}{\ddot{x}}_{2}\rangle =T_{e2}\left(\omega_{n}\right)-T_{f2}\left(\omega_{n}\right)+\delta m_{2}r_{2}\langle {\ddot{x}}_{2}\sin\varphi_{2}\rangle =\\ T_{e2}\left(\omega_{n}\right)-T_{f2}\left(\omega_{n}\right)+\delta m_{2}r_{2}\langle \left[-\mu_{21}\omega_{n}^{2}\cos\varphi_{1}-\mu_{22}\omega_{n}^{2}\cos\varphi_{2}\right]\sin\varphi_{2}\rangle =\\ T_{e2}\left(\omega_{n}\right)-T_{f2}\left(\omega_{n}\right)+\frac{1}{2}\delta m_{2}r_{2}\mu_{21}\omega_{n}^{2}\sin\left(\alpha_{1}-\alpha_{2}\right). \end{array}$$ (10)

Therefore,

$$\frac{1}{2}m_{1}r_{1}\mu_{12}\omega_{n}^{2}\sin\left(\alpha_{1}-\alpha_{2}\right)=\frac{1}{2}\delta m_{2}r_{2}\mu_{21}\omega_{n}^{2}\sin\left(\alpha_{1}-\alpha_{2}\right).$$ (11)

$$T_{v}=m_{1}r_{1}\mu_{12}\omega_{n}^{2}\sin\left(\alpha_{1}-\alpha_{2}\right)=\delta m_{2}r_{2}\mu_{21}\omega_{n}^{2}\sin\left(\alpha_{1}-\alpha_{2}\right).$$ (12)

where *T*_*v*_ is the vibration torque (VT) of the system. Rearranging Eqs (9) and (10), we have

$$P_{1}=T_{e1}\left(\omega_{n}\right)-T_{f1}\left(\omega_{n}\right)-\frac{1}{2}T_{v},$$ (13)

$$P_{2}=T_{e2}\left(\omega_{n}\right)-T_{f2}\left(\omega_{n}\right)+\frac{1}{2}T_{v}.$$ (14)

*T*_*v*_ acts on rotors as a driving force or resistance to regulate the energy distribution between the rotors. This is the key system index in realizing the vibration self-synchronization of the rotors. The value of *T*_*v*_ is related to the synchronous speed, the inertia moment of the rotors and the coupling spring. Subtracting Eq (14) from Eq (13), we have

$$P_{1}-P_{2}=T_{e1}\left(\omega_{n}\right)-T_{e2}\left(\omega_{n}\right)-\left[T_{f1}\left(\omega_{n}\right)-T_{f2}\left(\omega_{n}\right)\right]-T_{v}.$$ (15)

Introducing the following variable substitutions

$$\Delta \alpha =\alpha_{1}-\alpha_{2},\;\Delta T_{e}\left(\omega_{n}\right)=T_{e1}\left(\omega_{n}\right)-T_{e2}\left(\omega_{n}\right),\;\Delta T_{f}\left(\omega_{n}\right)=T_{f1}\left(\omega_{n}\right)-T_{f2}\left(\omega_{n}\right).$$ (16)

we can obtain

$$\sin\Delta \alpha =\left[\Delta T_{e}\left(\omega_{n}\right)-\Delta T_{f}\left(\omega_{n}\right)\right]\cdot \frac{1}{m_{2}r_{2}\omega_{n}^{2}\mu_{21}}.$$ (17)

The system synchronization index *D* is defined as

$$\frac{1}{D}=\left[\Delta T_{e}\left(\omega_{n}\right)-\Delta T_{f}\left(\omega_{n}\right)\right]\cdot \frac{1}{m_{2}r_{2}\omega_{n}^{2}\mu_{21}}.$$ (18)

Therefore,

$$\sin\Delta \alpha =\frac{1}{D}.$$ (19)

If Eq (17) has a real solution, the absolute value of its right side should be less than or equal to 1, and the synchronization condition for the system can be expressed as follows:

|D|≥1. (20)

According to references [1] and [16], the stability criterion of the synchronous state can be discussed based on Lyapunov stability theory.

$$P_{1}-P_{2}=\Delta T_{e}\left(\omega_{n}\right)-\Delta T_{f}\left(\omega_{n}\right)-T_{v}.$$ (21)

$$\frac{\partial \left(P_{1}-P_{2}\right)}{\partial \Delta \alpha }<0.$$ (22)

It can be deduced that

$$\frac{\delta }{\left(\omega_{1}^{2}+\eta \omega_{p}^{2}-\omega_{n}^{2}\right)\left(\omega_{2}^{2}+\omega_{p}^{2}-\omega_{n}^{2}\right)-\eta \omega_{p}^{4}}\cos\Delta \alpha >0.$$ (23)

Eq (23) is called the stability criterion of the self-synchronous state for the two rotors. The synchronous state is closely related to the directions of the rotors. When the two rotors rotate in the same or reverse direction, the phase difference Δ*α* would lie in different quadrants of the coordinate system.

The so-called vibratory synchronization transmission refers to the phenomenon in which two rotors can rotate synchronously even though one of two motors is shut down after synchronization occurs. The VT *T*_*v*_ acts on the rotors as a driving force or resistance. The coupling effect between the two rotors is strong enough that the VT *T*_*v*_ can overcome the resistance of one rotor after its motor is shut down. As vibratory synchronization transmission is obtained after the motor of rotor *i* is shut down, the corresponding synchronization index *D*_*Ti*_ can be expressed as

$$\frac{1}{D_{Ti}}=\left[{\left(-1\right)}^{i-1}T_{ei}\left(\omega_{n}\right)-\Delta T_{f}\left(\omega_{n}\right)\right]\cdot \frac{1}{m_{2}r_{2}\omega_{n}^{2}a_{21}}$$ (24)

Thus, as synchronization of the rotors occurs, only the motor of rotor *i* is turned on, and the condition that vibration synchronous transmission can be achieved is as follows:

$$\left|D_{Ti}\right|\geq 1$$ (25)

The motor state is not reflected in the stability criterion, so the stability criterion of vibratory synchronization transmission is still Eq (23).

## Discussions of theoretical results

The weakly damped system with two rotors mounted on different bodies is a coupled dynamic system consisting of two vibration subsystems. While the system satisfies the synchronization condition and the stability criterion, even if the vibration parameters of the motors, rotors and the vibrating bodies are obviously different, self-synchronous rotation of the rotors can still be achieved. Controlling the stiffness of the coupling spring can directly enhance or reduce the coupling between the rotors, so it is easy to adjust the synchronizing characteristics of the system by the coupling spring. In this paper, the two rotors rotate in the same direction, that is, *δ* = 1, and the other parameters of the system are shown in Table 1.

**Table 1:** Parameters of the system.

Parameters	Rotor 1	Rotor 2	Parameters	Rotor 1	Rotor 2
*M*_*i*_[kg]	300	200	*L*_*mi*_[H]	0.14	0.14
*m*_*i*_[kg]	3.5	2.5	*L*_*si*_ [H]	0.12	0.12
*j*_*i*_[kg·m^2^]	0.3	0.3	*R*_*ri*_[Ω]	0.6	0.6
*r*_*i*_[m]	0.15	0.1	*ω*_*si*_[rad/s]	314	314
*f*_*i*_[N·s/m]	200	200	*U*_*0*_[V]	220	220
*k*_*i*_[N/m]	7.5×10^5^	7.4×10^5^	*n*	2	2
*f*_*ri*_[N·m·s/rad]	3×10^−2^	1.47×10^−1^			

From Eqs (6) and (12), we have

$$T_{v\max}\left(\omega_{p}^{2}\right)=\frac{\delta m_{1}r_{1}m_{2}r_{2}\omega_{n}^{4}\omega_{p}^{2}}{M_{1}\left[\left(\omega_{1}^{2}-\omega_{n}^{2}\right)\left(\omega_{2}^{2}-\omega_{n}^{2}\right)+\left(\omega_{1}^{2}-\omega_{n}^{2}+\eta \left(\omega_{2}^{2}-\omega_{n}^{2}\right)\right)\omega_{p}^{2}\right]}.$$ (26)

where *T*_*vmax*_($\omega_{p}^{2}$) refers to the maximum vibration torque (MVT) of the system when the synchronous speed is *ω*_*n*_ and the stiffness of the coupling spring is *k*_*p*_. The equation *T*_*v*_ *= T*_*vmax*_($\omega_{p}^{2}$)sinΔ*α* can be obtained. Reference [1] and related information indicate that if rotational speeds of the two rotors are *ω*_*n1*_ and *ω*_*n2*_, when they are driven independently by motors, the synchronous speed should be between *ω*_*n1*_ and *ω*_*n2*_.

(1) The coupling spring *k*_*p*_ is small, and we Taylor expand MVT *T*_*vmax*_ by $\omega_{p}^{2}$ around 0.

$$T_{v\max}\left(\omega_{p}^{2}\right)=\frac{\delta m_{1}r_{1}m_{2}r_{2}\omega_{n}^{4}}{M_{1}\left(\omega_{1}^{2}-\omega_{n}^{2}\right)\left(\omega_{2}^{2}-\omega_{n}^{2}\right)}\cdot \omega_{p}^{2}+ο\left(\omega_{p}^{2}\right).$$ (27)

*T*_*vmax*_($\omega_{p}^{2}$) is proportional to $\omega_{p}^{2}$ in the first-order Taylor expansion. This indicates that when the coupling spring is small, the coupling between the rotors is weak, and it is difficult for rotors to self-synchronize.

(2) The coupling spring *k*_*p*_ increases gradually.

As we assume in section 2, the model is an after-resonance system and d*φ*_*1*_/d*t*, *φ*_*2*_/d*t* >2*ω*_*1*_, 2*ω*_*2*_.

For the after-resonance system, the denominator of the expression on the right-hand side of Eq (24) is zero when *ω*_*p*_ is a specific value *χ*, where

$$\chi^{2}=\frac{\left(\omega_{1}^{2}-\omega_{n}^{2}\right)\left(\omega_{2}^{2}-\omega_{n}^{2}\right)}{\omega_{n}^{2}-\omega_{1}^{2}+\eta \left(\omega_{n}^{2}-\omega_{2}^{2}\right)}.$$ (28)

**Fig 2:**
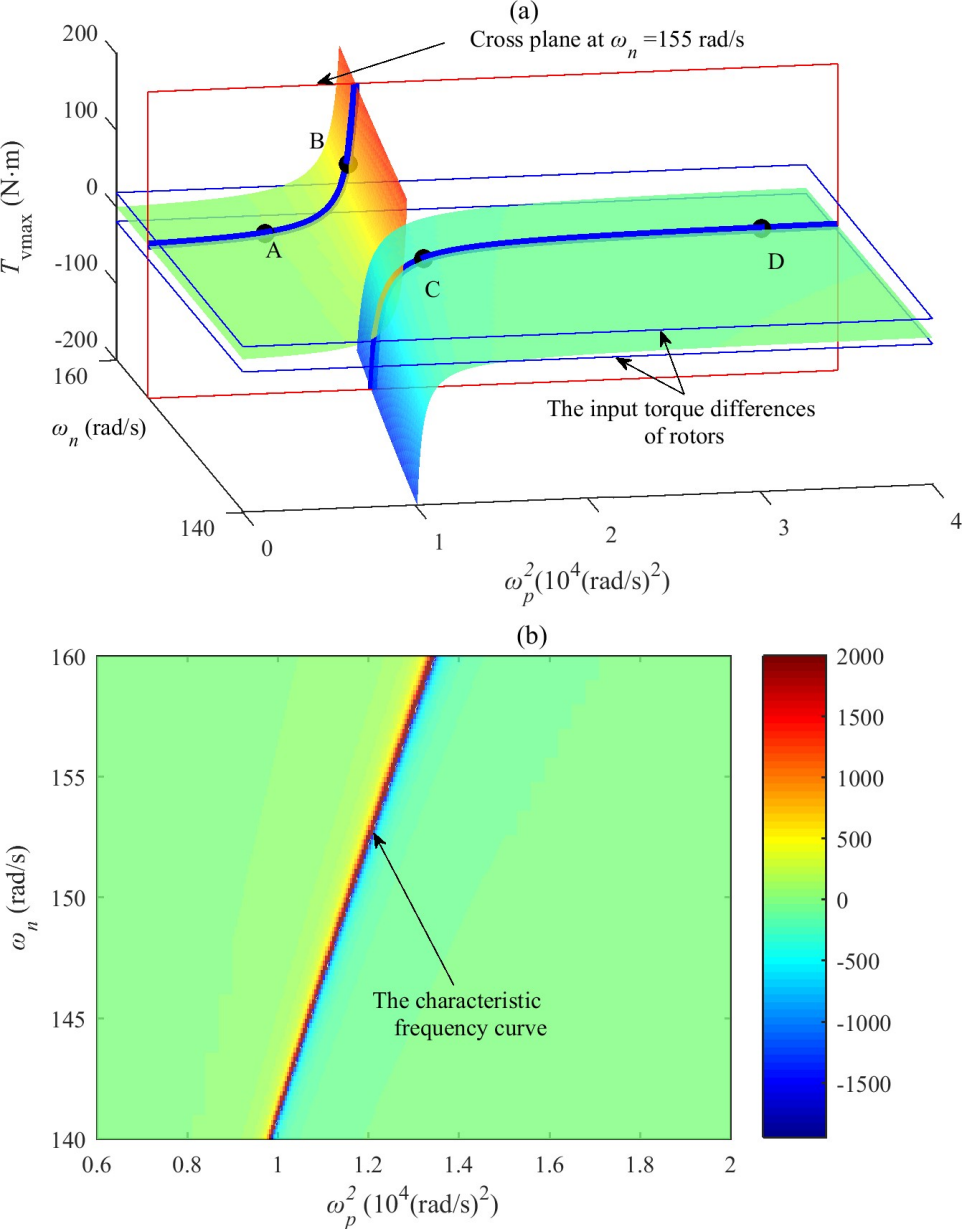
**Surface of the maximum vibration moment varying with the coupling frequency and synchronous speed in the after-resonance system** (a) The three-dimensional diagram. (b) The two-dimensional diagram.

**Fig 3:**
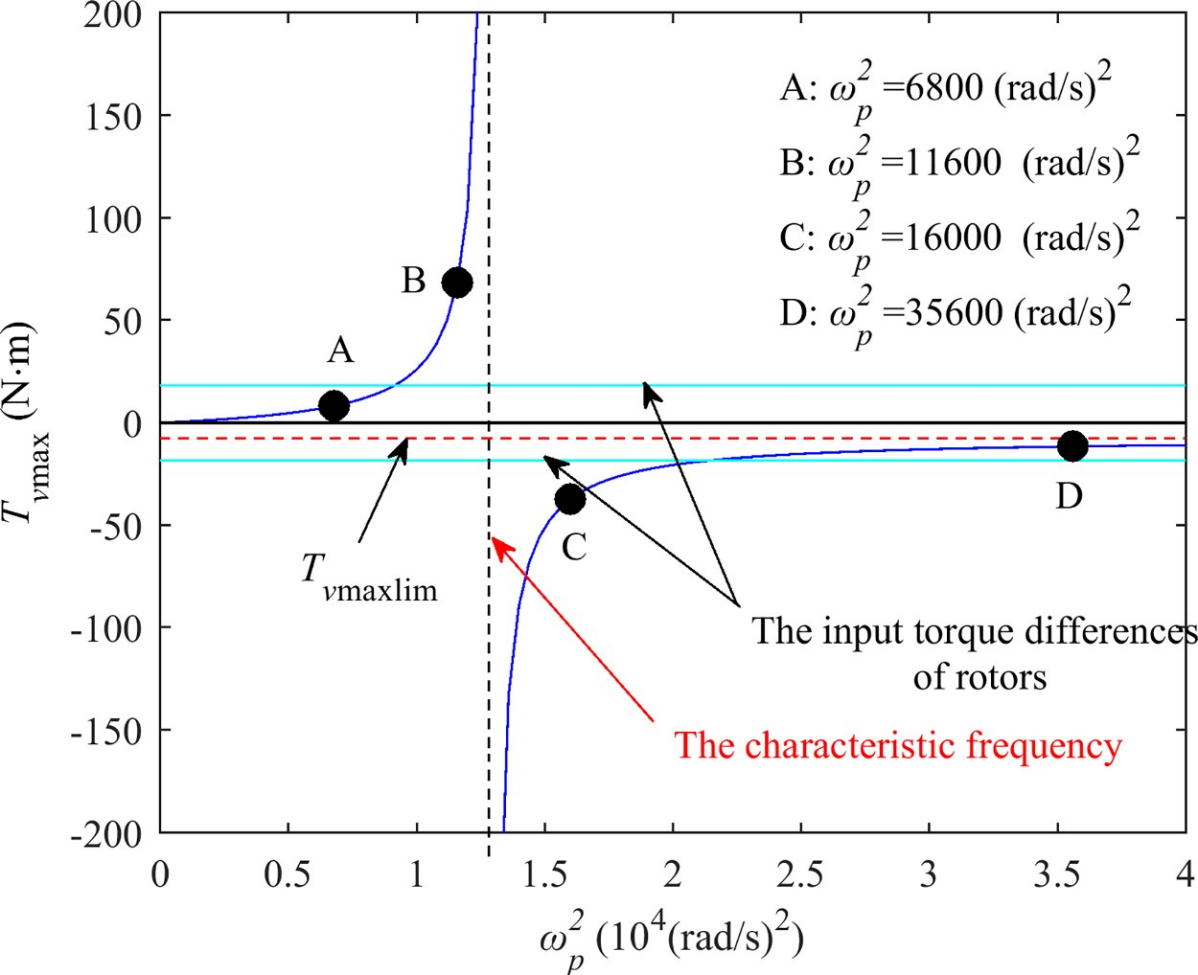
Relationship between the maximum vibration moment and coupling frequency when the synchronous speed is 155 rad/s in the after-resonance system.

As $\omega_{p}^{2}$ approaches *χ*^*2*^, *T*_*vmax*_($\omega_{p}^{2}$) tends to infinity. It can be assumed that the system tends to a resonant state. Therefore, *χ* is called the characteristic frequency (CF) of the system, as shown in Fig 2(A) and Fig 3. In the coordinates of *ω*_*n*_ and $\omega_{p}^{2}$, a curve consisting of characteristic frequencies at different synchronous speeds is called a characteristic frequency curve, as shown in Fig 2(B).

The curve of *T*_*vmax*_($\omega_{p}^{2}$) versus $\omega_{p}^{2}$ is shown in Fig 3 when *ω*_*n*_ is a particular value (for example, *ω*_*n*_ = 155 rad/s); it is the intersection line between the surface of Fig 2 and the cross plane passing through that particular value (*ω*_*n*_ = 155 rad/s). From Fig 2 and Eq (12), we can suggest that for different synchronous rotating speeds (such as between 140~160 rad/s), the curves of *T*_*vmax*_($\omega_{p}^{2}$) versus $\omega_{p}^{2}$ are all similar to that of Fig 2. The synchronous speed varies with the stiffness of the coupling spring and can be obtained by numerical simulation.

In Fig 3, the curve *T*_*vmax*_ consists of two parts, which are on the two sides of the CF *χ*. Approaching *χ*^*2*^ from the lower-frequency side, the MVT *T*_*vmax*_ tends to infinity; as $\omega_{p}^{2}$ increases past *χ*^*2*^ to infinity (i.e., the two vibrating bodies are fixed to each other), the MVT *T*_*vmax*_ gradually decreases and tends to a constant value *T*_*vmax*lim_, which is the maximum vibration torque that can be produced by the vibration system with two rotors mounted on the same body. Compared with the system in which the coupling frequency *ω*_*p*_ is close to the characteristic frequency *χ*, the coupling effect of the two rotors mounted on the same body is weaker.

In Fig 3, the curve *T*_*vmax*_ is divided into four parts by the curves of ±(Δ*T*_*e*_(*ω*_*n*_)_*2013*_Δ*T*_*f*_(*ω*_*n*_)), which are the input torque differences of the rotors. The four parts can be denoted by *LA* (passing through point A), *LB* (passing through point B), *LC* (passing through point C) and *LD* (passing through point D). According to Eqs (19) and (20), self-synchronization of the two rotors cannot be obtained when the system state occurs on *LA* and *LD* (i.e., |*T*_*vmax*_*|<|*Δ*T*_*e*_(*ω*_*n*_)_*–*_Δ*T*_*f*_(*ω*_*n*_)*|*). In contrast, the rotations of the two rotors can self-synchronize when the system state occurs on *LB* and *LC*.

Similarly, the surface of the MVT in Fig 2 is divided into four parts by the input torque difference planes of the rotors. The four parts are denoted by *SA* (passing through point A), *SB* (passing through point B), *SC* (passing through point C) and *SD* (passing through point D). Self-synchronization of the two rotors cannot be observed on the surfaces *SA* and *SD* but can be obtained on the surfaces *SB* and *SC*.

(3) When *ω*_*p*_→+∞, i.e. *k*_*p*_→+∞, we have

$$\underset{k_{p}\to +\infty }{\lim}\frac{1}{D}=\left[\Delta T_{e}\left(\omega_{n}\right)-\Delta T_{f}\left(\omega_{n}\right)\right]\cdot \frac{M\left(\omega_{1}^{2}+\omega_{2}^{2}-2\omega_{n}^{2}\right)}{2\delta m_{1}r_{1}m_{2}r_{2}\omega_{n}^{4}}.$$ (29)

We introduce *k*_*0*_ *= k*_*1*_*+k*_*2*_, $\omega_{0}^{2}$ = (*k*_*1*_*+k*_*2*_)/*M*, where *ω*_*0*_ is the natural frequency of the system when two vibrating bodies are fixed to each other. Eq (18) can be expressed as

$$\underset{k_{p}\to +\infty }{\lim}\frac{1}{D}=\left[\Delta T_{e}\left(\omega_{n}\right)-\Delta T_{f}\left(\omega_{n}\right)\right]\cdot \frac{k_{0}-M\omega_{n}^{2}}{\delta m_{1}r_{1}m_{2}r_{2}\omega_{n}^{4}}.$$ (30)

and Eq (19) can be expressed as

$$\sin\Delta \alpha =\left[\Delta T_{e}\left(\omega_{n}\right)-\Delta T_{f}\left(\omega_{n}\right)\right]\cdot \frac{k_{0}-M\omega_{n}^{2}}{\delta m_{1}r_{1}m_{2}r_{2}\omega_{n}^{4}}.$$ (31)

When *k*_*p*_→+∞, the stability criterion of self-synchronization can be deduced as follows:

$$\frac{\delta }{k_{0}-M\omega_{n}^{2}}\cos\Delta \alpha >0.$$ (32)

As shown in Figs 2 and 3, for the after-resonance system, when the system coupling frequency *ω*_*p*_ is close to the characteristic frequency *χ* or the system state is near the characteristic frequency curve, the system coupling performance is strong, and self-synchronization of the two rotors can be obtained easily. It is convenient to control the synchronization performance by adjusting the coupling spring stiffness *k*_*p*_. In the after-resonance system, the coupling performance between the two rotors mounted on the same rigid body is not the strongest. Therefore, when it is difficult for two rotors mounted on the same vibrating body self-synchronize, it may be effective to install the two rotors on a flexible body with a certain stiffness or on two vibrating bodies connected by a coupling spring. Looser self-synchronization conditions can be obtained, and the frequency capture performance of the system is better.

## Simulations for the synchronization of two rotors

The model discussed in this paper is a weakly damped nonresonant vibrating system with two rotors mounted on different bodies. Dynamic simulations of the system are carried out with $\omega_{p}^{2}$ set to 6800 (rad/s)^2^, 11600 (rad/s)^2^, 16000 (rad/s)^2^, 35600 (rad/s)^2^ and infinity (i.e., *k*_*p*_ is equal to 1.36×10^6^ N/m, 2.32×10^6^ N/m, 3.2×10^6^ N/m, 7.12×10^6^ N/m and tends to infinity), corresponding to points A, B, C and D. In Table 1, the differences between the two rotors and the two motors are obvious. Rotor 2 is subjected to a larger damping force than rotor 1 with the same rotational speed. The simulations are carried out under this condition.

**Fig 4:**
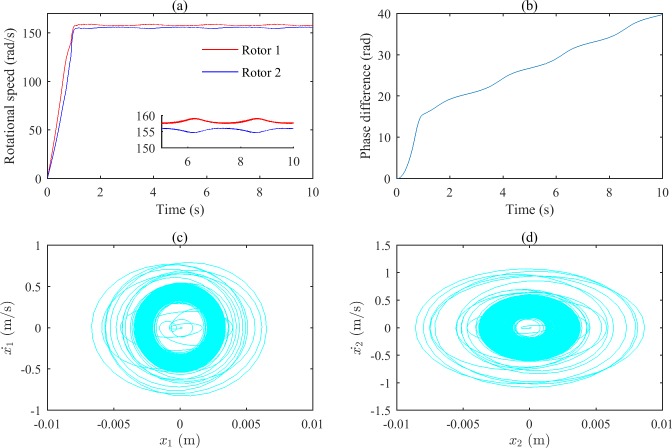
Simulation results of the after-resonance system when the coupling stiffness is 1.36×10^6^ N/m. (a) Rotational speeds of the two rotors. (b) The phase difference between the two rotors. (c) The phase portrait of body 1.(d) The phase portrait of body 2.

Fig 4 shows the simulation results when $\omega_{p}^{2}$ is equal to 6800 (rad/s)^2^. From Figs 2 and 3, we can suggest that the rotations of the two rotors would self-synchronize in these states. As shown in Fig 4(A), the speeds of the two rotors are not consistent when the system reaches stable conditions; Fig 4(B) demonstrates that the phase difference of the two rotors increases gradually as a result of the asynchrony. Fig 4(C) and 4(D) show that the fluctuations of the two bodies’ phase portraits are obvious.

**Fig 5:**
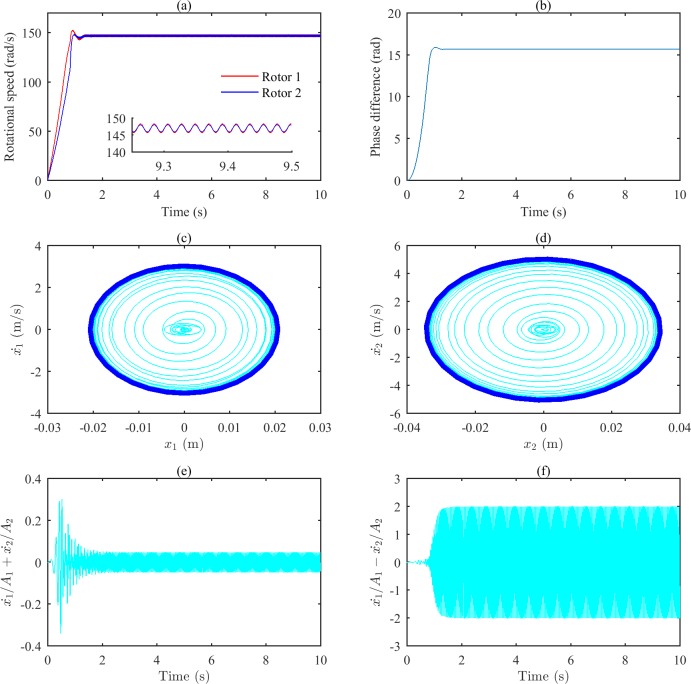
Simulation results of the after-resonance system when the coupling stiffness is 2.32×10^6^ N/m. (a) Rotational speeds of the two rotors. (b) The phase difference between the two rotors. (c) The phase portrait of body 1.(d) The phase portrait of body 2. (e) Sum of two bodies’ relative displacements. (f) Difference of two bodies’ relative displacements.

When the rotors run synchronously, the two bodies vibrate regularly. Under this condition, the amplitudes of the two bodies 1 and 2 are denoted by *A*_*1*_ and *A*_*2*_, respectively. We can briefly investigate the relative position of the two bodies by *x*_*1*_*/A*_*1*_*+x*_*2*_*/A*_*2*_ and *x*_*1*_*/A*_*1–*_*x*_*2*_*/A*_*2*_. Fig 5 shows the simulation results when $\omega_{p}^{2}$ is set to 11600 (rad/s)^2^, which is close to the characteristic frequency. According to Figs 2 and 3, the MVT |*T*_*vmax*_| is larger than the input torque difference of the rotor *|*Δ*T*_*e*_(*ω*_*n*_)_*–*_Δ*T*_*f*_(*ω*_*n*_)*|* under this condition, so there is a real solution for Eq (19). The value of Δ*α* is calculated to be 3.28 rad based on Eqs (16) and (22). As shown in Fig 5(A), the speeds of the two rotors reach the same value at approximately 1.5 s, and Fig 5(B) shows that the phase difference of the two rotors is stable at 15.83 rad (15.83–4×π = 3.26(rad)). The dark blue curves in Fig 5(C) and 5(D) indicate the same movement of the vibrating bodies with different vibration periods. From Fig 5(E) and 5(F), it can be obtained that the two bodies vibrate in roughly the same direction.

**Fig 6:**
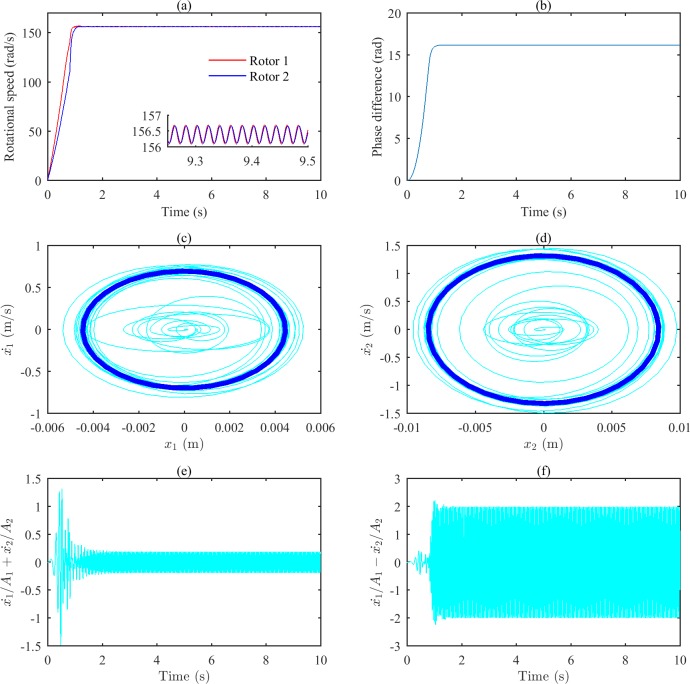
Simulation results of the after-resonance system when the coupling stiffness is 3.20×10^6^ N/m. (a) Rotational speeds of the two rotors. (b) The phase difference between the two rotors. (c) The phase portrait of body 1.(d) The phase portrait of body 2. (e) Sum of two bodies’ relative displacements. (f) Difference of two bodies’ relative displacements.

Fig 6 shows the simulation results when $\omega_{p}^{2}$ is set to 16000 (rad/s)^2^. Similar to the simulation results with $\omega_{p}^{2}$ set to 11600 (rad/s)^2^, the parameters of the system satisfy the self-synchronization conditions in this state. The value of Δ*α* is calculated to be 3.65 rad based on Eq (19). As shown in Fig 6(A), the speeds of the two rotors reach the same value at approximately 1.5 s, and Fig 6(B) indicates that the phase difference of the two rotors is stable at 16.21 rad (16.21–4×π = 3.64(rad)). The numerical results shown in Figs 5 and 6 are consistent with the theoretical analysis. The same movement of vibrating bodies in different vibration periods is presented by the dark blue curves in Fig 6(C) and 6(D). Similarly, in Fig 6(E) and 6(F), the movements of the two bodies are generally in the same direction as well.

**Fig 7:**
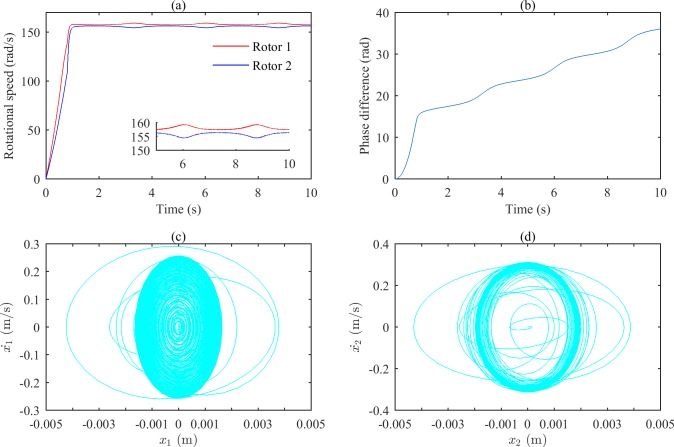
Simulation results of the after-resonance system when the coupling stiffness is 7.12×10^6^ N/m. (a) Rotational speeds of the two rotors. (b) The phase difference between the two rotors. (c) The phase portrait of body 1.(d) The phase portrait of body 2.

**Fig 8:**
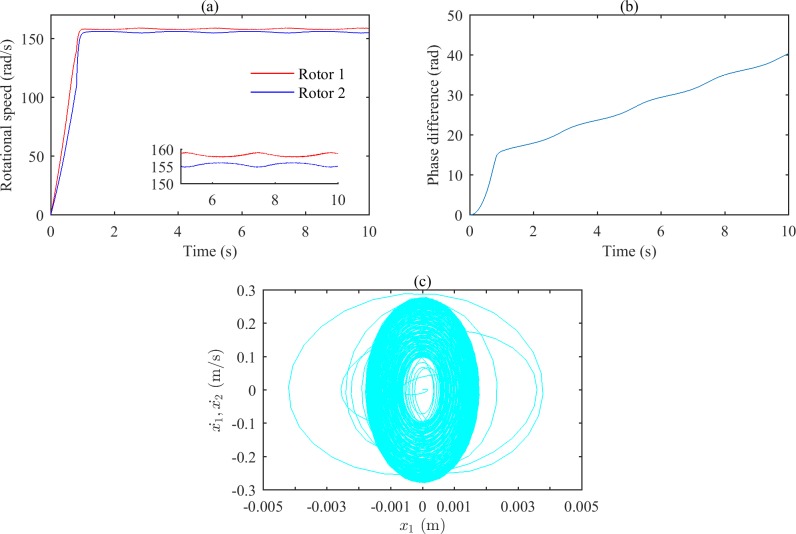
Simulation results of the after-resonance system when the coupling stiffness tends to infinity. (a) Rotational speeds of the two rotors. (b) The phase difference between the two rotors. (c) The phase portrait of bodies 1and 2.

Figs 7 and 8 show the simulation results when $\omega_{p}^{2}$ is set to 35600 (rad/s)^2^ and tends to infinity, respectively. According to Figs 2 and 3, the rotations of the two rotors would self-synchronize under those conditions. As shown in Figs 7(A) and 8(A), the speeds of the two rotors are not consistent when the system reaches stable conditions; Figs 7(B) and 8(B) demonstrate the asynchrony of the two rotors. Figs 7(C), 7(D), 8(C) and 8(D) show that the vibrations of the two bodies are irregular.

The simulation results show that in the after-resonance system, the coupling effects of the system will be strong when the coupling frequency is close to the characteristic frequency, and self-synchronization of the two rotors occurs easily. When there is a large difference between the coupling frequency and the characteristic frequency, it will be difficult to achieve self-synchronization.

## Simulations for vibratory synchronization transmission of two rotors

**Fig 9:**
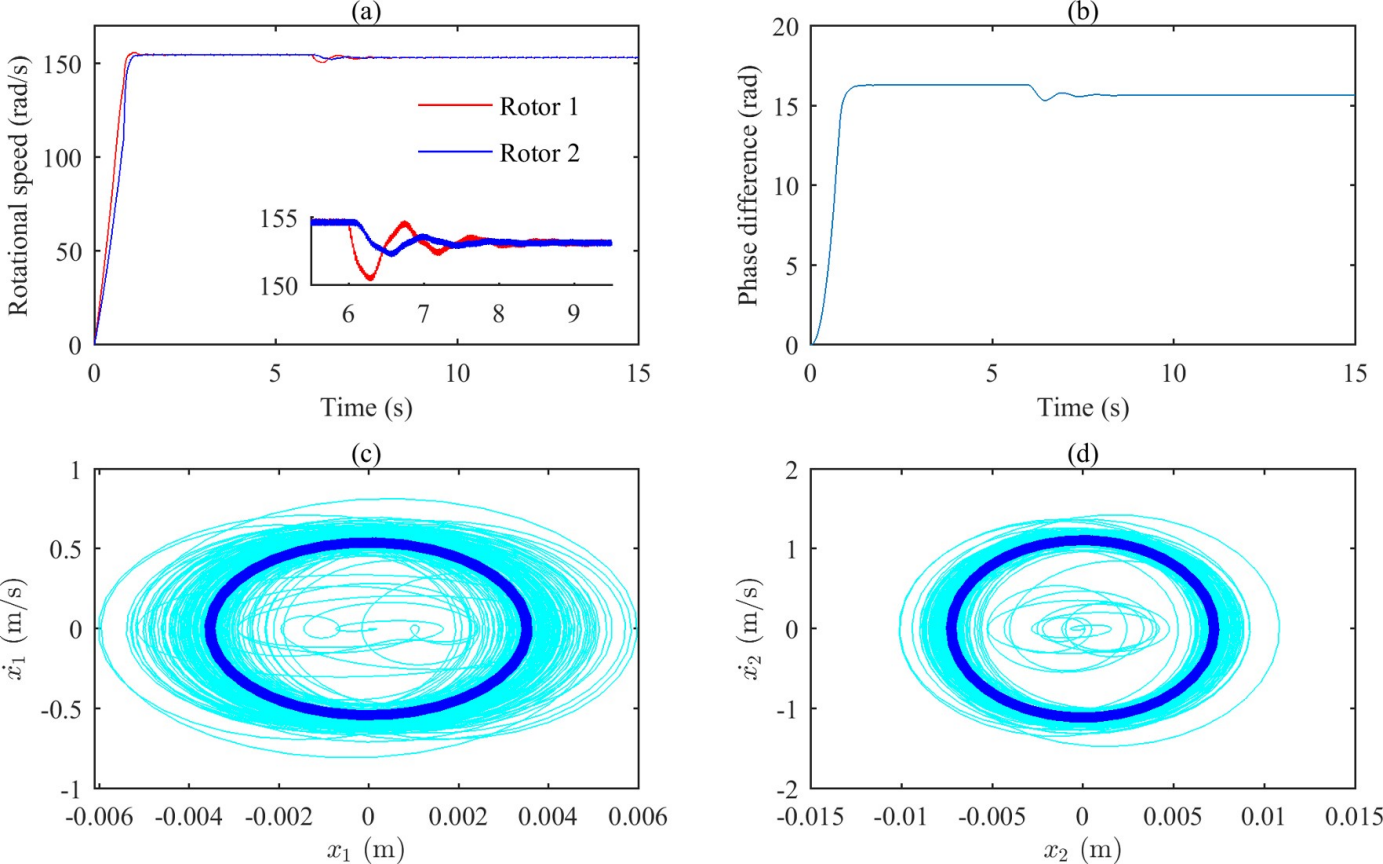
Simulation results of the system with motor 1 removed. (a) Rotational speeds of the two rotors. (b) The phase difference between the two rotors. (c) The phase portrait of body 1.(d) The phase portrait of body 2.

**Fig 10:**
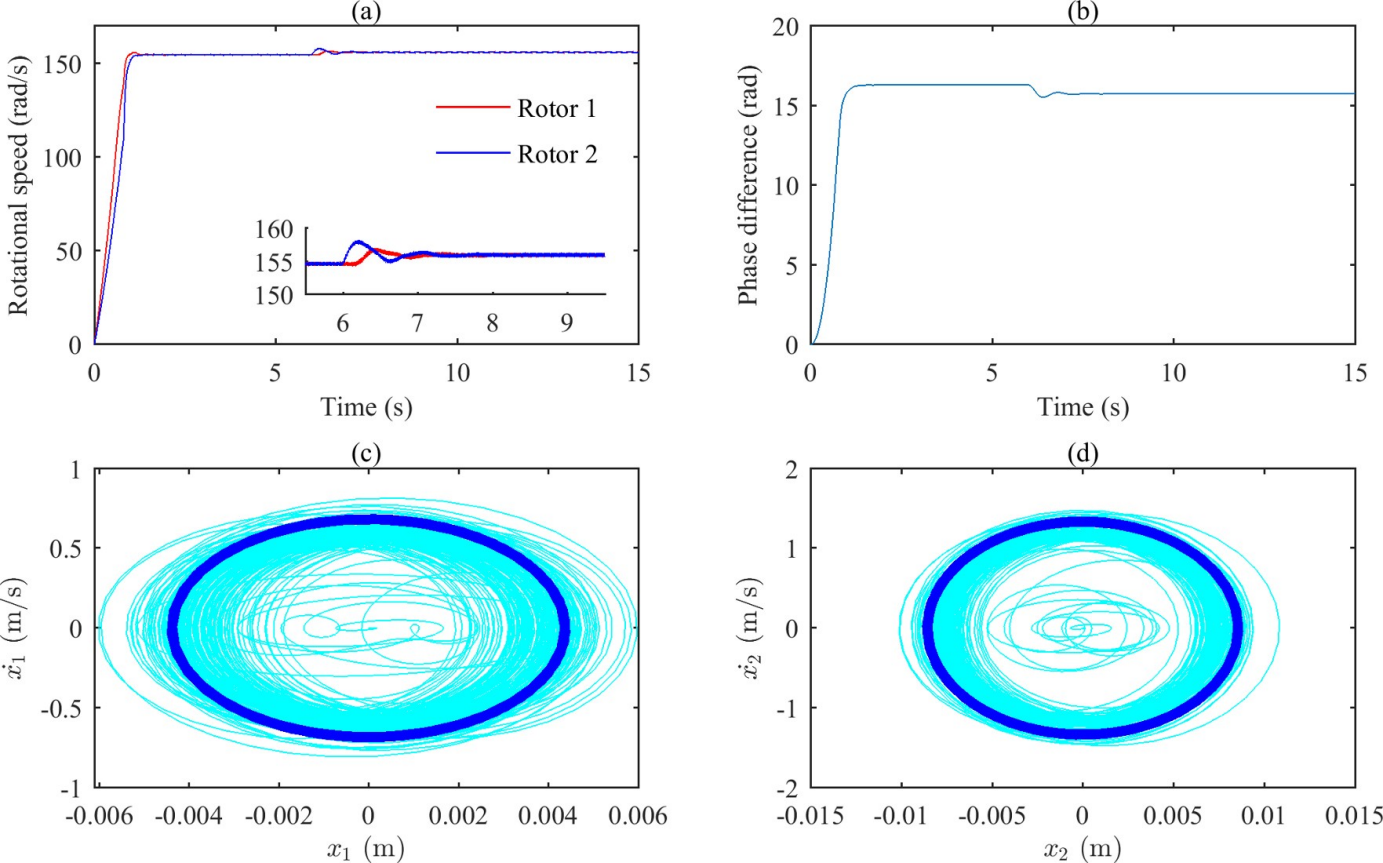
Simulation results of the system with motor 2 removed. (a) Rotational speeds of the two rotors. (b) The phase difference between the two rotors. (c) The phase portrait of body 1.(d) The phase portrait of body 2.

According to the above research, when $\omega_{p}^{2}$ is 16000 (rad/s)^2^, synchronization of the two rotors can be obtained. Based on this state, vibratory synchronization transmission of the system is studied. As the system vibrates stably, the motors of rotor 1 and rotor 2 are removed, and the simulation results of vibratory synchronization transmission are shown in Fig 9 and Fig 10.

Fig 9 demonstrates that the rotations of the two rotors synchronize at approximately 2 s, and the synchronous speed is approximately 154.67 rad/s. Although motor 1 is removed at 6 s, the two rotors can still rotate synchronously because of the coupling effects between the rotors. Δ*α* changes to 15.66 rad (15.74–4π = 3.09) from 16.30 rad (16.30–4π = 3.73). With *ω*_*n*_ = 153.15 rad/s, *T*_*vmax*_ = –29.17 N, and Δ*T*_*e*_(*ω*_*n*_)–Δ*T*_*f*_(*ω*_*n*_) = –0.6162 N, the system can meet the synchronous criterion indicated by Eq (24). Fig 10 represents the simulation of the system with motor 2 removed at 6 s. From Fig 10, we can see that the two rotors can still be synchronized due to the coupling effect despite the disappearance of motor 2. Δ*α* changes to 15.74 rad (15.74–4π = 3.17) from 16.30 rad (16.30–4π = 3.73). With *ω*_*n*_ = 155.99 rad/s, *T*_*vmax*_ = –34.85 N, and Δ*T*_*e*_(*ω*_*n*_)–Δ*T*_*f*_(*ω*_*n*_) = 1.41 N, the system can satisfy the criterion of vibratory synchronization transmission. The dark blue curves in Figs 9(C), 9(D), 10(C) and 10(D) indicate the stable movement of the vibrating bodies after vibratory synchronization transmission of the two rotors is obtained.

For the synchronization of the two rotors, the VT *T*_*v*_ balances the energy input difference between the two rotors and acts as a driving force (or resistance) on the backward rotor (the leading rotor). The motor of the leading rotor not only outputs energy to maintain the rotation of the rotor but also provides energy to the vibrating system. If the coupling effect is strong enough, rotor *i* can still rotate and synchronize with the other rotor despite the shutdown of motor *i*. This means that the vibration of the system provides the energy required for the rotor to maintain rotation. It should be noted that as the motor *i* is shut down and vibratory synchronization transmission is achieved, both the synchronous speed and phase difference between rotors change, and the system adopts a new synchronization condition.

## Conclusions

Self-synchronization of two rotors can occur in a weakly damped nonresonant vibrating system with two rotors mounted on different bodies. The synchronization condition is that the vibration torque is large enough to overcome the input torque difference of the two rotors, and the synchronization state should satisfy the stability criterion. If the coupling effect of the system is strong enough, vibratory synchronization transmission can be achieved when one of the two motors is shut off.

1. Synchronization of the two rotors is sensitive to the system parameters. For the after-resonance system, there is a characteristic frequency or a characteristic frequency curve. The coupling effects of the system can be strong when the coupling frequency is close to the characteristic frequency, and self-synchronization of the two rotors can occur easily. When there is a large difference between the coupling frequency and the characteristic frequency, self-synchronization will not be achieved.
2. For the after-resonance system, the coupling performance of the two rotors will be weakened as the coupling frequency *ω*_*p*_ is increased above the characteristic frequency *χ* towards infinity (two rotors are installed on the same vibrating body). Looser self-synchronization conditions for the system can be obtained by controlling the stiffness of the coupling spring. Designed according to this principle, the synchronization of vibrating systems or dual-rotor exciters can be achieved with good stability and high tolerance.
3. As vibratory synchronization transmission is achieved, the vibration of the system provides the energy required for the rotor to maintain rotation after its motor is shut down. Both the synchronous speed and phase difference between the rotors change due to the removal of the motor, and the system approaches a new synchronization state.

## Supporting information

S1 Dataset (RAR)
